# Emission Sector Impacts on Air Quality and Public Health in China From 2010 to 2020

**DOI:** 10.1029/2021GH000567

**Published:** 2022-06-01

**Authors:** Luke Conibear, Carly L. Reddington, Ben J. Silver, Ying Chen, Stephen R. Arnold, Dominick V. Spracklen

**Affiliations:** ^1^ Institute for Climate and Atmospheric Science School of Earth and Environment University of Leeds Leeds UK; ^2^ College of Engineering, Mathematics and Physical Sciences University of Exeter Exeter UK

**Keywords:** emulators, air quality, China, machine learning, health impact assessment, emissions

## Abstract

Anthropogenic emissions and ambient fine particulate matter (PM_2.5_) concentrations have declined in recent years across China. However, PM_2.5_ exposure remains high, ozone (O_3_) exposure is increasing, and the public health impacts are substantial. We used emulators to explore how emission changes (averaged per sector over all species) have contributed to changes in air quality and public health in China over 2010–2020. We show that PM_2.5_ exposure peaked in 2012 at 52.8 μg m^−3^, with contributions of 31% from industry and 22% from residential emissions. In 2020, PM_2.5_ exposure declined by 36% to 33.5 μg m^−3^, where the contributions from industry and residential sources reduced to 15% and 17%, respectively. The PM_2.5_ disease burden decreased by only 9% over 2012 where the contributions from industry and residential sources reduced to 15% and 17%, respectively 2020, partly due to an aging population with greater susceptibility to air pollution. Most of the reduction in PM_2.5_ exposure and associated public health benefits occurred due to reductions in industrial (58%) and residential (29%) emissions. Reducing national PM_2.5_ exposure below the World Health Organization Interim Target 2 (25 μg m^−3^) would require a further 80% reduction in residential and industrial emissions, highlighting the challenges that remain to improve air quality in China.

## Key Points

- We used emulators of long‐term air quality in China to estimate the emission sector impacts on air pollution and health over 2010–2020
- PM_2.5_ exposure peaked in 2012 at 52.8 μg m^−3^, primarily from industrial (31%) and residential (22%) emissions
- PM_2.5_ exposure reduced by 36% in 2020, reducing the disease burden by 9% mostly from lower industrial (58%) and residential (29%) emissions

## 1 Introduction

Long−term ambient fine particulate matter (PM_2.5_) and ozone (O_3_) exposure is a leading public health problem in China, associated with 9% (95% uncertainty interval, 95UI: 7–11%) of the healthy life lost to disease in 2019 (GBD, 2019 Risk Factors Collaborators, 2020; Yin et al., 2020). Previous studies have found that long−term ambient PM_2.5_ exposure (population−weighted concentrations) in China peaked over 2011–2013 at between 50 and 65 μg m^−3^ (Geng et al., 2021; Health Effects Institute, 2020; Huang et al., 2021; Liang et al., 2020; Silver, Conibear, et al., 2020; Yue et al., 2020; Q. Zhang et al., 2019). Since 2013, PM_2.5_ exposure has decreased at a rate of 0.5–5 μg m^−3^ yr^−1^, reaching 35–48 μg m^−3^ in 2019 (Geng et al., 2021; Health Effects Institute, 2020; Huang et al., 2021; Kong et al., 2021; Liang et al., 2020; Ma et al., 2019; McDuffie et al., 2021; Silver, Conibear, et al., 2020; Xue et al., 2019; Yue et al., 2020; Zhai et al., 2019; Zhang et al., 2019). In contrast, O_3_ exposure increased over 2014–2017 (Health Effects Institute, 2020; K. Li et al., 2019; Lu et al., 2020; Silver, He, et al., 2020).

The reductions in PM_2.5_ concentrations over 2015–2017 have been attributed to reductions in anthropogenic emissions, as a result of the Air Pollution Prevention and Control Action Plan of 2013–2017 (Cheng et al., 2019; Ding et al., 2019; K. Li et al., 2019, 2020; Silver, Conibear, et al., 2020; Zhai et al., 2019; B. Zhao et al., 2018). The plan required PM_2.5_ concentrations to decline by 25% in Beijing–Tianjin–Hebei, 20% in the Yangtze River Delta, and 15% in the Pearl River Delta. A range of emission controls were implemented including tighter emission standards for industry, power generation, and land transport, in addition to phasing out inefficient industrial plants (B. Zheng et al., 2018). Reduced use of residential solid fuels, primarily due to urbanization and increased incomes rather than specific policies, also improved particulate air quality (B. Zhao et al., 2018; H. Zheng et al., 2019). Bottom−up estimates of anthropogenic emissions changes over 2015–2017 suggest reductions in industrial (19%), residential (16%), and power generation (11%) emissions (B. Zheng et al., 2018). The previous studies of sector contributions to air quality in China mainly analyze individual years over 2010–2020 using different methods (Gao et al., 2019; GBD MAPS Working Group, 2016; Gu et al., 2018; Hu, Huang, et al., 2017; Lelieveld et al., 2015; J. Liu, Xing, et al., 2020; McDuffie et al., 2021; C. Reddington et al., 2019). An ensemble of these estimates finds these contributions to be 30% from industry, 26% from residential, 16% from agricultural, 14% from power generation, and 7% from land transport emissions (C. Reddington et al., 2019). Despite the recent reductions in emissions and PM_2.5_ concentrations in China, PM_2.5_ exposure remains high, O_3_ exposure is increasing, and the associated disease burden is substantial (Conibear, Reddington, Silver, Knote, et al., 2021; Silver, Conibear, et al., 2020; B. Zhao et al., 2018).

Atmospheric models are useful for simulating air quality, estimating emission sector contributions, and exploring processes and mechanisms. However, their complexity comes at high computational costs, which limits the feasible number of experiments. One solution to this problem is to train emulators to learn statistical associations using data from these atmospheric models. The emulators are then much cheaper to run, substantially increasing the number of experiments that can be explored. Emulators are often designed using Gaussian processes (O’Hagan, 2006; Rasmussen & Williams, 2006). A key benefit of these is their accuracy with smaller data sets, which is useful here as the training data is simulated using the complex atmospheric models.

In Conibear, Reddington, Silver, Chen, et al. (2021), we developed the emulator approach for predicting short−term (1−month) PM_2.5_ exposure from emission changes in China (averaged per sector over all species). In Conibear, Reddington, Silver, Chen, Knote, et al. (2022), we extended the emulator approach for long−term (annual) exposure, multiple air pollutants (PM_2.5_ and O_3_), chronic health impacts, and explored the sensitivity of exposure and health to different combinations of emission reductions from a 2015 baseline. Here, we use these long−term emulators to explore how air quality and public health has changed in China over 2010–2020, how different emission sectors have contributed to these changes, and how it may continue to change beyond 2020. We combine the emulators with surface observations of air pollutants to produce new estimates of emission changes that we compare with existing studies.

## 2 Methods

### 2.1 Emulators and Simulators of Air Quality

We trained emulators to predict air quality across China from emission changes using simulation data. Simulations of air quality in China used WRFChem (Weather Research and Forecasting model online–coupled with Chemistry) version 3.7.1 (Grell et al., 2005; Skamarock et al., 2008). WRFChem was described and evaluated in our previous work (Conibear, Reddington, Silver, Chen, et al., 2021; Conibear, Reddington, Silver, Knote, et al., 2021; C. Reddington et al., 2019; Silver, Conibear, et al., 2020). The simulations were for 2015 at 30 km horizontal resolution. There were 50 simulations for the training data and 5 additional simulations for the test data. Each simulation only varied the fraction of anthropogenic emissions for these five sectors. The fractions were applied for each sector individually, determined using maxi−min Latin hypercube space–filling designs separately for both the training data and the test data. The scaling factors for each anthropogenic emission sector of the training and test simulators are provided in the Supplementary Tables of Conibear, Reddington, Silver, Chen, Knot, et al. (2022). This design produces efficient and accurate emulators, as demonstrated in our previous work (Y. Chen et al., 2020; Conibear, Reddington, Silver, Chen, et al., 2021; Conibear, Reddington, Silver, Chen, Knote, et al., 2022).

Anthropogenic emissions were from the MEIC (Multi–resolution Emission Inventory for China) emission inventory (M. Li, Liu, et al., 2017; M. Li, Zhang, et al., 2017; MEIC Research Group & Tsinghua University, 2019; Zheng et al., 2018). Gas phase chemistry was from the extended MOZART (Model for Ozone and Related Chemical Tracers) scheme (Emmons et al., 2010; Hodzic & Jimenez, 2011; Knote et al., 2014). Aerosol physics and chemistry were from the updated MOSAIC (Model for Simulating Aerosol Interactions and Chemistry) scheme with aqueous chemistry (Alma Hodzic & Knote, 2014; Zaveri et al., 2008). Secondary organic aerosol formation was from an updated volatility basis set mechanism (Knote et al., 2015).

The emulators were Gaussian process machine learning models, developed and evaluated in (Conibear, Reddington, Silver, Chen, et al., 2021; Conibear, Reddington, Silver, Chen, Knote, et al., 2022). The inputs to the emulators were fractional changes in anthropogenic emissions from the residential (RES), industrial (IND), land transport (TRA), agricultural (AGR), and power generation (ENE) sectors. The outputs of the emulators were the metrics used in the health impact assessment of annual−mean PM_2.5_ concentrations and maximum 6−monthly−mean daily−maximum 8−hour (6mDM8h) O_3_ concentrations. The 6mDM8h metric was calculated by quantifying 24 separate 8−hour rolling mean O_3_ concentrations, finding the maximum of these each day, creating 12 separate 6−monthly means to account for seasonal variations, and finding the maximum of these over the year.

There was one emulator per output and grid cell in China, with 30,556 emulators in total. The emulators predicted air quality for all emission configurations within a 0–150% matrix of emission scaling factors at 20% increments, with 32,768 emission configurations in total. The emulators were specific to their training data, and predicted based on associational knowledge, rather than explanatory knowledge (Deutsch, 2012; Pearl, 2019).

For the simulator evaluation (Figure 1 in Supporting Information S1), we independently assessed a control simulation against measurements across China (Jin et al., 2020; Silver et al., 2018). The control simulation underestimated PM_2.5_ concentrations (normalized mean bias factor, NMBF = −0.05 and normalized mean absolute error factor, NMAEF = 0.18) and overestimated O_3_ concentrations (NMBF = 0.39 and NMAEF = 0.40). In order to provide the closest match with observations, we scaled PM_2.5_ and O_3_ concentrations to measurements. We applied the scaling to the control model and applied identical scalings to the emulators. Scalings were applied by prefecture where observations were available, otherwise scalings were applied by province (administrative division). After this scaling was applied, the control simulation had low bias and error for both PM_2.5_ concentrations (NMBF = 0.02 and NMAEF = 0.10) and O_3_ concentrations (NMBF = 0.03 and NMAEF = 0.11). Our approach relies on the sensitivity of the WRFChem simulations to emissions change. Future work is needed to explore the sensitivity of concentrations simulated by WRFChem to uncertainty in the chemistry and physics of the model.

For the emulators evaluation (Figure 2 in Supporting Information S1), we independently assessed the (scaled) emulators on the unseen test simulations. The emulators accurately predicted the unseen test simulation data, with a coefficient of determination (R^2^) value for both PM_2.5_ and O_3_ concentrations of 0.999 and root mean squared errors (RMSE) of 0.5094 μg m^−3^ for PM_2.5_ and 0.1667 ppb for O_3_ concentrations. These evaluations showed that the simulators accurately represented the spatial pattern and magnitude of measured PM_2.5_ and O_3_ concentrations across China, and that the emulators accurately predicted the simulator (Conibear, Reddington, Silver, Chen, et al., 2021; Conibear, Reddington, Silver, Chen, Knote, et al., 2022).

The emulators were designed to quickly predict air quality solely from fractional changes in the five key anthropogenic emission sectors. The emulators did not account for changes in other sectors and sources due to computational constraints (Conibear, Reddington, Silver, Chen, Knote, et al., 2022). The simulated training data for the emulators was based on meteorology from 2015 and did not account for the interannual variation in meteorology over 2010–2020. The recent impacts from interannual changes in meteorology on air quality at the annual scale in China have been found to be substantially smaller than the impacts from changes in emissions (Ding et al., 2019; Silver, Conibear, et al., 2020; Zhang et al., 2019). We note that variability in meteorology can have a very important impact on air quality at shorter (days−weeks) timescales (Hammer et al., 2020).

### 2.2 Health Impact Assessment

The health impact assessment estimated the disease burden attributable to PM_2.5_ and O_3_ exposure using population attributable fractions (PAF) of relative risk (RR). Exposure variations were used to predict associated outcome variations.

The chronic PM_2.5_ disease burden was estimated using the GEMM (Global Exposure Mortality Model, Burnett et al., 2018). The outcomes were non−accidental mortality (non−communicable disease, NCD, plus lower respiratory infections, LRI). The minimum exposure of no excess risk was 2.4 μg m^−3^. The chronic O_3_ disease burden was estimated using the methods of the Global Burden of Diseases, Injuries, and Risk Factors Study (GBD) for 2017 (GBD 2017 Risk Factor Collaborators, 2018). The outcome was chronic obstructive pulmonary disease (COPD). The minimum exposure of no excess risk was 35.7 ppb (Turner et al., 2016).

The measures used were the number of premature mortalities (MORT) per year. The population count for 2010, 2015, and 2020 was from the Gridded Population of the World, Version 4.11, at 15 arc−minute resolution (Center for International Earth Science Information Network & NASA Socioeconomic Data and Applications Center, 2018). The population count was interpolated between these three years for the remaining years within 2010–2020. The population age groupings for 2010–2019 for adults of 25–80 years of age in 5−year intervals and for 80 years plus were from the GBD2019 (GBD 2019 Risk Factors Collaborators, 2020). The baseline health rates for 2010–2019 for each outcome (NCD: group category B, LRI: specific category A.2.2, and COPD: specific category B.3.1), measure, and age grouping were from the GBD2019 (Institute for Health Metrics and Evaluation, 2020). The population age groupings and baseline health rates were extrapolated for 2020.

Sector−specific changes in the air pollution disease burden can either be calculated using the subtraction or attribution methods (Conibear et al., 2018; Kodros et al., 2016). The subtraction method estimates the change in the air pollution disease burden over time. The attribution method estimates the sector−specific contributions to the air pollution disease burden. In high−exposure regions, the sector−specific public health benefits from the subtraction method are smaller than those from the attribution method due to the non−linear exposure−outcome association for PM_2.5_ concentrations (Conibear et al., 2018; Kodros et al., 2016). Both of these approaches were in the results for their different purposes and were identified when used.

Shapefiles were used to aggregate results at the country, province, and prefecture level (Hijmans et al., 2020). Uncertainty intervals at the 95% confidence level were estimated using the uncertainty intervals from the exposure−outcome associations, baseline health rates, and population age groupings. Health impact assessments of the disease burden associated with air pollution exposure have many uncertainties (Nethery & Dominici, 2019). These include uncertainties in the simulator (i.e., in WRFChem from input data, parameterisations, grid aggregations, etc.), exposure−outcome associations (e.g., confounding, induction, study variability), and population generalisations (e.g., non−representative cohorts, extrapolations). The uncertainties and limitations of the population count data are detailed in Center for International Earth Science Information Network & NASA Socioeconomic Data and Applications Center, (2018). Present day emissions in China are uncertain, especially for non−methane volatile organic compounds (VOC) emissions (M. Li et al., 2018; M. Li, Liu, et al., 2017; Saikawa et al., 2017; Zhong et al., 2019).

### 2.3 Measurement−Informed and Bottom−Up Emission Changes

We combined the emulators with measurements over the last 6 years to derive a “measurement−informed” estimate of the change in emissions that occurred over this period. The measured changes in annual−mean PM_2.5_ and 6mDM8h O_3_ concentrations over 2015–2020 were calculated for each measurement station (1,633 in total). The year of 2015 was chosen as the start year, as this is when extensive measurements became available. Each measurement station was spatially paired to the nearest emulator. The emission configurations were taken at 20% increments, with the edges of the parameter space (0% and 140%) removed (7,776 emission configurations remaining). The predicted changes in air quality were calculated for the remaining emission configurations relative to the baseline. The measured and predicted changes in air quality were compared, and those that matched within 1% were retained. The emission configurations that corresponded to these retained predictions were then counted to find the most frequently occurring. The top 1,000 occurring emission configurations were analyzed for matching trends in PM_2.5_ concentrations only, due to the emulators ability to accurately capture the trend in PM_2.5_ concentrations. These most common emission configurations represented measurement−informed estimates of the changes in anthropogenic emissions that matched the measured trend in air quality. We do not account for the impacts of interannual variability in meteorology, though we expect that this has relatively small impacts at the annual scale.

The measurement−informed estimate was compared to the bottom−up estimate of the changes in anthropogenic emissions over 2010–2017 from Zheng et al. (2018) (Table 1 in Supporting Information S1). Mean emission changes were calculated over carbon monoxide, nitrogen oxides (NO_X_), sulfur dioxide, ammonia (NH_3_), black carbon, organic carbon, PM_2.5_, coarse particulate matter, and VOC emissions. The results were similar when averaging over a few key species (i.e., NO_X_, VOC, NH_3_, and PM_2.5_) instead of averaging over all species. The results using the average over all species were presented here, as the emulator inputs were averaged over all species. The bottom−up emission change estimates from Zheng et al. (2018) used sector−specific MEIC emissions from 2010–2017. MEIC emissions cover 31 provinces in China and include approximately 700 anthropogenic sources.

## 3 Results and Discussion

In the results and discussion, PM_2.5_ concentrations are ambient annual−means and O_3_ concentrations are ambient 6mDM8h. Exposures are population−weighted concentrations.

### 3.1 Measurement−Informed and Bottom−Up Emission Estimates Over 2015−2017

**Figure 1:**
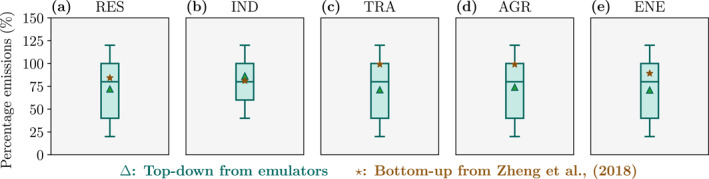
Comparison of our estimates of anthropogenic emission changes in China over 2015–2017 with previous and bottom−up estimates (B. Zheng et al., 2018). Our estimates are for the top 1,000 occurring emission configurations that matched the measured trend in fine particulate matter (PM_2.5_, annual−mean) concentrations only. Emissions are for the (a) residential (RES), (b) industrial (IND), (c) land transport (TRA), (d) agricultural (AGR), and (e) power generation (ENE) sectors. Boxplot percentiles are fifth, 25th, 50th, 75th, and 95th. Mean emission changes are over carbon monoxide, nitrogen oxides, sulfur dioxide, ammonia, black carbon, organic carbon, PM_2.5_, coarse particulate matter, and non−methane volatile organic compounds.

The bottom−up emissions for 2015–2017 from Zheng et al. (2018) suggest large reductions in industrial (19%), residential (16%), and power generation (11%) emissions (Figure 1). Our measurement−informed estimate for 2015–2017 suggest larger reductions in power generation (29%) and residential (28%) emissions and smaller reductions in industrial (14%) emissions. The reductions in land transport (29%) and agricultural (26%) emissions from our estimate are larger than the bottom−up reductions (both 1%).

The measurement−informed estimate has large variability, where many possible emission configurations match the measured trends in air quality. We calculate different mean emission reductions if we match to PM_2.5_ concentrations only, O_3_ concentrations only, both PM_2.5_ and O_3_ concentrations, or either PM_2.5_ or O_3_ concentrations (Figures 3–5 in Supporting Information S1). We note that our approach well matches the observed trend in PM_2.5_ concentrations, but does not well match the observed trend in O_3_ concentrations (see Section 3.2), providing larger confidence in emission estimates based on PM_2.5_ concentrations. If our estimate is matched to both PM_2.5_ and O_3_ concentrations, then there are larger reductions in industry of 21% (Figure 4 in Supporting Information S1). If our estimate is matched to O_3_ concentrations only, then the reductions are larger in power generation (57%), agriculture (36%), and industry (24%), and smaller in residential (18%) emissions (Figure 5 in Supporting Information S1). The reductions in land transport emissions are similar for all matching methods.

### 3.2 Trends in Emissions, Exposure, and Public Health Over 2010−2020

**Figure 2:**
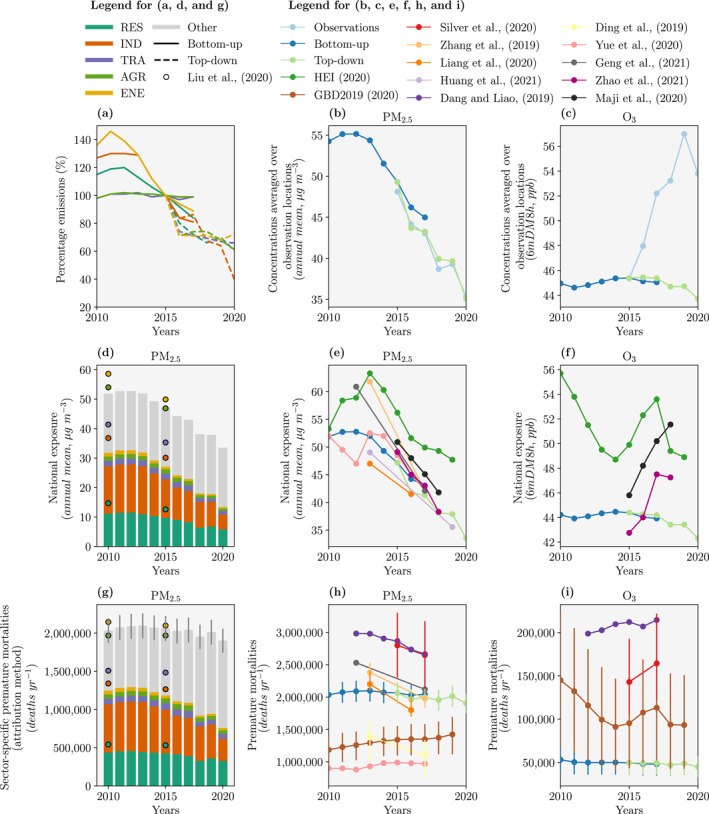
Changes in emissions, air quality, and health impacts in China over 2010–2020. The bottom−up estimates use emissions from Zheng et al. (2018) for 2010–2017. The measurement−informed estimates are from the emulators for 2015–2020 using the mean of the top 1,000 occurring emission configurations that match the measured trend in fine particulate matter (PM_2.5_, annual−mean) concentrations only. Results are for (a) mean emission changes relative to 2015 across all species, (b) mean PM_2.5_ concentrations at observation locations, (c) mean ozone (O_3_, maximum 6−monthly−mean daily−maximum 8−hour, 6mDM8h) concentrations at observation locations, (d) sectoral contributions to PM_2.5_ exposure, (e) national PM_2.5_ exposure, (f) national O_3_ exposure, (g) sector−specific premature mortalities (MORT) from PM_2.5_ exposure using the attribution method, (h) annual MORT from PM_2.5_ exposure, and (i) annual MORT from O_3_ exposure. Sectors are residential (RES), industrial (IND), land transport (TRA), agricultural (AGR), and power generation (ENE) emissions. Results are added from various previous studies (Dang & Liao, 2019; Ding et al., 2019; GBD 2019 Risk Factors Collaborators, 2020; Geng et al., 2021; Health Effects Institute, 2020; Huang et al., 2021; Liang et al., 2020; J. Liu, Xing, et al., 2020; Maji & Sarkar, 2020; Silver, Conibear, et al., 2020; Yue et al., 2020; Q. Zhang et al., 2019; H. Zhao et al., 2021).

Figure 2 shows 2010–2020 changes in emissions, air quality, and health impacts over China. Bottom−up emissions are from B. Zheng et al. (2018). These emissions are combined with our emulators to produce bottom−up concentrations, exposure, and disease burden. Our measurement−informed emission estimates are the mean emission configurations that when combined with the emulator match the trend in measured concentrations (matched to PM_2.5_ concentrations only). The concentrations from these simulations are used to calculate measurement−informed exposure and disease burden. Our measurement−informed emission estimates were compared with bottom−up estimates for 2015–2017 when both estimates were available.

In the bottom−up estimate, power generation emissions steeply decline over 2011–2016, and slightly decrease further to 2020 in the measurement−informed estimate (Figure 2a). Industrial and residential emissions both decrease from 2013–2017 in the bottom−up estimate, and both further decrease in 2020 compared to 2017 in the measurement−informed estimate. Land transport and agricultural emissions remain relatively unchanged in the bottom−up estimate over 2010–2017, while decrease after 2015 in the measurement−informed estimate.

From 2015–2020, average observed PM_2.5_ concentrations at measurement locations reduced by 12.7 μg m^−3^ (Figure 2b), well reproduced by our estimates (−14.2 μg m^−3^). Over 2015–2017, the observed change in PM_2.5_ concentrations (−5.1 μg m^−3^), is slightly underestimated by the bottom−up estimate (−4.3 μg m^−3^) and slightly overestimated by the measurement−informed estimate (−6.0 μg m^−3^). From 2017–2020, observed PM_2.5_ concentrations reduce by 7.7 μg m^−3^ (Figure 2b), similar to the reduction in the measurement−informed estimate (8.2 μg m^−3^). The regional trends for PM_2.5_ exposure are similar for all regions (Figure 6 in Supporting Information S1).

Average PM_2.5_ exposure across China declined by 36% from a peak of 52.8 μg m^−3^ (bottom−up estimate) in 2012 to 33.5 μg m^−3^ (measurement−informed estimate) in 2020 (Figure 2e). However, we note this compares estimates from bottom−up and measurement−informed estimates. The reasonable agreement between these two approaches over 2015–2017 when both are available suggests this comparison is appropriate. In the bottom−up estimate, national PM_2.5_ exposure increases from 2010 (51.9 μg m^−3^) to 2012 (52.8 μg m^−3^), and then decreases by 5.6 μg m^−3^ over 2012–2015 (a reduction of 11%, Figure 2e). A similar reduction in national PM_2.5_ exposure over 2013–2019 was seen in previous work (Geng et al., 2021; Health Effects Institute, 2020; Huang et al., 2021; Liang et al., 2020; Maji & Sarkar, 2020; Silver, Conibear, et al., 2020; Yue et al., 2020; Q. Zhang et al., 2019; H. Zhao et al., 2021).

From 2015–2017, observed O_3_ concentrations increased by 6.9 ppb (Figure 2c) compared to small changes in both bottom−up (−0.3 ppb) and measurement−informed (−0.0 ppb) estimates. The inability of the model to simulate the observed trend means that simulated national O_3_ exposure, which remains relatively constant over 2010–2015 at 44 ppb (Figure 2f), is unlikely to be realistic. From 2017–2020, observed O_3_ concentrations increase by a further 1.6 ppb (Figure 2c), while the measurement−informed estimate slightly decreases by 1.7 ppb. The decrease in measurement−informed O_3_ exposure over 2017–2020 is driven by reductions over South Central, South West, and North West China, while measurement−informed O_3_ exposure increases in North China (Figure 6 in Supporting Information S1).

As the measurement−informed estimates of O_3_ concentrations do not match the observed positive trend in O_3_ concentrations (Figure 2c), we are likely to underestimate the increase in disease burden associated with O_3_ exposure over 2010–2020. The bottom−up and measurement−informed estimates may not match the observed trend in O_3_ concentrations in China because of emission uncertainties over China (M. Li, Liu, et al., 2017; Saikawa et al., 2017) or due to missing chemistry in the simulators (K. Li et al., 2020, 2021). Previous work found that meteorology variability had a substantially smaller impact on the O_3_ concentration trend than either emissions or chemistry, and so is unlikely to explain the mismatch (K. Li et al., 2019, 2021). Some previous studies found a different trend in national O_3_ exposure, where it increased from 2014 to 2017 (Health Effects Institute, 2020; Maji & Sarkar, 2020; H. Zhao et al., 2021).

In our analysis, the PM_2.5_ disease burden declines by 9% from a peak of 2,091,100 (95UI: 1,925,000–2,252,000) premature deaths per year in 2012 (bottom−up estimate) to 1,903,300 (95UI: 1,745,100−2,057,800) premature deaths per year in 2020 (measurement−informed estimate, Figure 2h). This is less than the concurrent 36% decline in PM_2.5_ exposure (Figure 2e), due to the non−linear exposure−outcome association and population aging.

Most previous studies find a reduction in PM_2.5_ disease burden due to reduced PM_2.5_ exposure (Ding et al., 2019; Geng et al., 2021), with a larger trend in studies that did not account for population aging (Dang & Liao, 2019; Liang et al., 2020; Silver, Conibear, et al., 2020; Q. Zhang et al., 2019). We estimate that if population age, count, and baseline health were kept constant at 2012 rates, then the PM_2.5_ disease burden in 2020 would have reduced by 22% relative to 2012. The population count in China grew by 5% over 2010–2020. If the population count did not increase beyond 2010 levels, then we estimate that the disease burden in 2020 would have been 7% smaller.

In contrast, some previous studies found that the impact on the disease burden from population aging outweighed that from exposure reductions. For example, the GBD 2019 Risk Factors Collaborators (2020) found that the PM_2.5_ disease burden increased by 13% over 2012–2019 and Yue et al. (2020) found that the PM_2.5_ disease burden increased by 10% over 2012–2017. The increasing disease burden in these studies, in comparison to the reducing disease burden in this study, is primarily due to their smaller exposure reductions of 19% for GBD 2019 Risk Factors Collaborators, (2020) and 10% for Yue et al. (2020).

Based on the bottom−up emissions, the national disease burden associated with O_3_ exposure in 2010 is 53,200 (95UI: 38,400−67,700) premature deaths per year (Figure 2i). This disease burden remains approximately the same in the bottom−up (until 2017) and measurement−informed (until 2020) estimates. Previous studies found the national disease burden from O_3_ exposure increased over 2015–2017, primarily from increased exposure (Dang & Liao, 2019; GBD 2019 Risk Factors Collaborators, 2020; Silver, Conibear, et al., 2020). The health impact assessments in previous studies use a wide range of exposure estimation methods, exposure−outcome associations, and input data.

### 3.3 Emission Sector Contributions to Exposure Over 2010−2020

**Figure 3:**
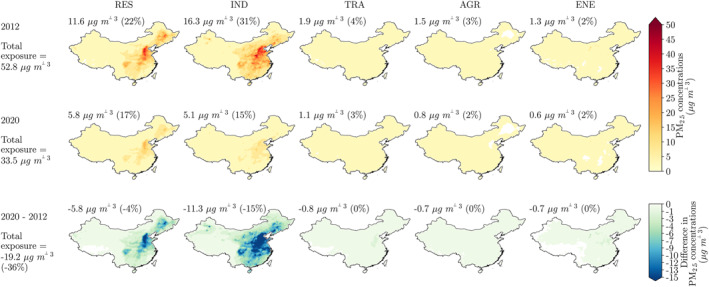
Sectoral contributions to ambient fine particulate matter (PM_2.5_, annual−mean) concentrations and exposure in China for 2012, 2020, and 2020 minus 2012. The results for 2012 are the bottom−up estimate using emissions from Zheng et al. (2018). The results for 2020 are the measurement−informed estimate from the emulators using the mean of the top 1,000 occurring emission configurations that match the measured trend in PM_2.5_ concentrations only. The rows are for the years of 2012, 2020, and 2020 minus 2012. The row annotations (far left) show the PM_2.5_ exposure for these years, with the percentage change for 2020 minus 2012. The columns are for the PM_2.5_ concentrations per sector of residential (RES), industrial (IND), land transport (TRA), agricultural (AGR), and power generation (ENE) emissions. The columns annotations (above each map) show the absolute and percentage attribution of PM_2.5_ exposure to that sector for 2012 and 2020, and the percentage change between these for 2020 minus 2012.

At the PM_2.5_ exposure peak in 2012 (bottom−up) of 52.8 μg m^−3^, the sector contributions are 31% from industrial, 22% from residential, 4% from land transport, 3% from agriculture, and 2% from power generation emissions (Figure 3). Over 2012 (bottom−up) to 2020 (measurement−informed), when PM_2.5_ exposure decreased by 36% to 33.5 μg m^−3^, the contributions reduce in industry to 15% (−15% points) and in residential to 17% (−4% points), while remaining approximately the same in land transport, agriculture, and power generation emissions (Figure 3). The PM_2.5_ exposure reductions are largest in East and North China. The substantial reduction in PM_2.5_ concentrations from industrial sources over 2012–2020 (−11.3 μg m^−3^) means that residential emissions make the largest contribution to PM_2.5_ exposure in 2020, despite the 5.8 μg m^−3^ reduction in this sector.

Industrial, residential, energy generation, land transport, and agriculture emissions together contributed 62% of PM_2.5_ exposure in 2012 (bottom−up), declining to 40% in 2020 (measurement−informed). This means the contribution to PM_2.5_ exposure from other sources increased from 38% to 60% over 2012–2020 (Figure 2d). These other sources include other anthropogenic sources inside China such as shipping, aviation, and agricultural fires, anthropogenic emissions outside China, and natural emission sources. Previous studies have estimated that dust contributes up to 10% of PM_2.5_ concentrations in China (McDuffie et al., 2021; Shi et al., 2017; Yang et al., 2011), waste combustion up to 9% (McDuffie et al., 2021), fires up to 8% (C. Reddington et al., 2019; C. L. Reddington et al., 2021; Shi et al., 2017), biogenic secondary organic aerosol (SOA) up to 8% (Hu, Wang, et al., 2017; Shi et al., 2017), anthropogenic emissions outside China up to 3% (S. Liu, Xing, et al., 2020), shipping up to 3% (C. Chen et al., 2019; Dasadhikari et al., 2019; McDuffie et al., 2021; C. Reddington et al., 2019), aviation up to 1% (Dasadhikari et al., 2019; Zhang et al., 2017), and sea salt up to 1% (Shi et al., 2017). This would suggest the importance of other anthropogenic emission sources inside China (21% including fire as an anthropogenic source) and natural emissions (19%), with a smaller contribution from anthropogenic sources outside China (3%).

We estimate the reduction in PM_2.5_ exposure over 2012–2020 results in 187,800 (95UI: 179,900−194,200) fewer premature deaths per year in 2020 (subtraction method). Most of these public health benefits are from reductions in industrial emissions (58%), then residential emissions (29%), with smaller contributions from reductions in land transport (4%), agriculture (3%), and power generation (3%) emissions.

J. Liu, Xing, et al. (2020) found that the sector attributions to PM_2.5_ concentrations over 2010–2015 reduced in industry from 38% to 35% and in power generation from 8% to 6%, increased in agriculture from 22% to 23%, and remained the same in residential emissions at 25% (Figure 2d). We find a similar decrease in the attribution to industry (from 31% to 28%), while the attribution to other sectors remained approximately the same (residential at 21%, land transport at 4%, agriculture at 3%, and power generation at 2%). The PM_2.5_ disease burden from J. Liu, Xing, et al. (2020) was the total of the contributions from the power, industry, residential, transportation, and agriculture sectors, and did not include other sources. J. Liu, Xing, et al. (2020) found that most of the reduction in the PM_2.5_ disease burden were attributed to reductions in industrial emissions with 63,700 (95UI: 55,800−70,400) fewer premature deaths (attribution method), similar to our estimate of 110,100 (95UI: 105,500−113,900) fewer premature deaths (Figure 2g).

We estimate that national PM_2.5_ exposure could meet the World Health Organization (WHO) Interim Target 2 (25 μg m^−3^) (World Health Organization, 2021) by reducing residential and industrial emissions by 80% below 2020 emissions (equivalent to a 88–92% reduction in 2015 emissions). Regional PM_2.5_ exposure varies under this scenario, where it is lower in the Greater Bay Area (16.2 μg m^−3^) and South West China (17.1 μg m^−3^), and higher in North China (30.2 μg m^−3^). These emissions reductions would reduce the 2020 national disease burden associated with PM_2.5_ exposure by 23%, avoiding a further 440,800 (95UI: 424,200–444,500) premature deaths each year. The WHO Interim Target 2 (25 μg m^−3^) is the lowest attainable target from changes solely in these five emission sectors. Removing emissions from the five sectors in China does not enable the attainment of the WHO Annual Guideline (5 μg m^−3^) due to the remaining emissions from shipping, aviation, waste combustion, and agricultural fires, emissions from outside China, and emissions from natural sources including forest fires, mineral dust, and vegetation (biogenic SOA).

## 4 Conclusion

We used emulators to explore how different emission sectors have contributed to air quality and public health changes in China over 2010–2020. We show that national PM_2.5_ exposure peaked in 2012 at 52.8 μg m^−3^, then declined by 36% to 33.5 μg m^−3^ in 2020. The associated PM_2.5_ disease burden declined from 2,091,100 (95UI: 1,925,000–2,252,000) premature deaths in 2012 to 1,903,300 (95UI: 1,745,100−2,057,800) premature deaths in 2020. This 9% reduction (187,800, 95UI: 179,900−194,200, fewer premature deaths per year in 2020) would have been larger if it were not for an aging population. Most of these public health benefits are from reduced industrial (58%) and residential (29%) emissions. The contribution from other sources to PM_2.5_ exposure increases from 38% to 60% over 2012–2020.

Our work highlights the challenges faced by China to further improve air quality and public health. Despite the National Air Quality Target (35 μg m^−3^) being met at the national level in 2020, the disease burden from PM_2.5_ exposure remains substantial. Reducing national mean PM_2.5_ exposure below the WHO Interim Target 2 (25 μg m^−3^) would require 80% reductions in both residential and industrial emissions, which would avoid 440,800 (95UI: 424,200–444,500) premature deaths each year. China has implemented strategies to reduce emissions in the power generation, industrial, and land transportation sectors, achieving large reductions in PM_2.5_ exposure. However, the recent strategy for tackling residential emissions has focused on North China (Meng et al., 2020). The expansion of these strategies to South China could provide substantial health benefits (Conibear, Reddington, Silver, Knote, et al., 2021). For example, removing residential emissions could reduce PM_2.5_ exposure by 22% in South West China, 19% in South Central China, and 8% in the Greater Bay Area (GBA). Our work emphasizes the importance of further reductions in industrial and residential emissions and the need for policy to address a broader range of pollution sources.

## Conflict of Interest

The authors declare no conflicts of interest relevant to this study.

## Supporting information

Supporting Information S1

## Data Availability

Code to setup and run WRFChem (using WRFotron version 2.0) is available through Conibear and Knote (2020). Emulator code and data is available through Conibear (2021). The trained emulators per grid cell in China that support the findings of this study are available in Conibear, Reddington, Silver, Chen, Arnold, et al. (2022).
