# Supplementary material for: Emission Sector Impacts on Air Quality and Public Health in China From 2010 to 2020
Source: Geohealth. 2022 Jun 1;6(6):e2021GH000567. doi: 10.1029/2021GH000567 (PMC9207900; doi:10.1029/2021GH000567)
Supplement: Supplementary file 1 — Supporting Information S1 [file GH2-6-e2021GH000567-s001.pdf]

Supporting information for

Emission Sector Impacts on Air Quality and Public Health in China from 2010−2020

Luke Conibear[*,1], Carly L. Reddington[1], Ben J. Silver[1], Ying Chen[2], Stephen R. Arnold[1], and Dominick V. Spracklen[1]

[1] Institute for Climate and Atmospheric Science, School of Earth and Environment, University of Leeds, Leeds, UK

[2] College of Engineering, Mathematics and Physical Sciences, University of Exeter, UK

* Corresponding author: Luke Conibear (L.A.Conibear@leeds.ac.uk)

Contents

Supplementary Table 1: The bottom−up emission configurations in China over 2010−2017 relative to 2015 from Zheng et al., (2018). The emission sectors are residential (RES), industry (IND), land transport (TRA), agriculture (AGR), and power generation (ENE). Mean emission changes over carbon monoxide, nitrogen oxides, sulphur dioxide, ammonia, black carbon, organic carbon, fine particulate matter, coarse particulate matter, and non−methane volatile organic compounds.

Supplementary Figure 1: Evaluation of unscaled and scaled baseline simulator air quality concentrations (Conibear et al., 2022). Regional evaluation metrics are normalised mean bias factor (NMBF) grouped by prefecture if available, otherwise by province for (a) for fine particulate matter (PM$_{2.5}$, annual−mean) and (d) ozone (O_3, maximum 6−monthly−mean daily−maximum 8−hour, 6mDM8h). Unscaled baseline concentrations for (b) PM$_{2.5}$ (NMBF = −0.05, normalised absolute error factor, NMAEF, = 0.18) and (e) O_3 (NMBF = 0.39, NMAEF = 0.40). Scaled baseline concentrations for (c) PM$_{2.5}$ (NMBF = 0.02, NMAEF, = 0.10) and (f) O_3 (NMBF = 0.03, NMAEF = 0.11).

Supplementary Figure 2: Evaluation of emulators on the unseen test data for concentrations of (a) fine particulate matter (PM$_{2.5}$, annual−mean) and (b) ozone (O_3, maximum 6−monthly−mean daily−maximum 8−hour, 6mDM8h) (Conibear et al., 2022). Evaluation metrics used were the coefficient of determination (R^2) and the root mean squared error (RMSE).

Supplementary Figure 3: Comparison of top−down (emulators) and bottom−up (Zheng et al., 2018) estimates of anthropogenic emission changes in China over 2015−2017. Top−down estimates are for the top 1,000 occurring emission configurations that matched the measured trend in *either fine particulate matter (PM$_{2.5}$, annual−mean) and ozone (O_3, maximum 6−monthly−mean daily−maximum 8−hour, 6mDM8h) concentrations*. Emissions are for the (a) residential (RES), (b) industrial (IND), (c) land transport (TRA), (d) agricultural (AGR), and (e) power generation (ENE) sectors. Boxplot percentiles are 5th, 25th, 50th, 75th, and 95th. Mean emission changes are over carbon monoxide, nitrogen oxides, sulphur dioxide, ammonia, black carbon, organic carbon, PM$_{2.5}$, coarse particulate matter, and non−methane volatile organic compounds.

Supplementary Figure 4: Comparison of top−down (emulators) and bottom−up (Zheng et al., 2018) estimates of anthropogenic emission changes in China over 2015−2017. Top−down estimates are from the top 1,000 occurring emission configurations that match the measured trend in *both fine particulate matter (PM$_{2.5}$, annual−mean) and ozone (O_3, maximum 6−monthly−mean daily−maximum 8−hour, 6mDM8h) concentrations*. Emissions are for the (a) residential (RES), (b) industrial (IND), (c) land transport (TRA), (d) agricultural (AGR), and (e) power generation (ENE) sectors. Boxplot percentiles are 5th, 25th, 50th, 75th, and 95th. Mean emission changes are over carbon monoxide, nitrogen oxides, sulphur dioxide, ammonia, black carbon, organic carbon, PM$_{2.5}$, coarse particulate matter, and non−methane volatile organic compounds.

Supplementary Figure 5: Comparison of top−down (emulators) and bottom−up (Zheng et al., 2018) estimates of anthropogenic emission changes in China over 2015−2017. Top−down estimates are for the top 1,000 occurring emission configurations that matched the measured trend *in ozone (O_3, maximum 6−monthly−mean daily−maximum 8−hour, 6mDM8h) concentrations only*. Emissions are for the (a) residential (RES), (b) industrial (IND), (c) land transport (TRA), (d) agricultural (AGR), and (e) power generation (ENE) sectors. Boxplot percentiles are 5th, 25th, 50th, 75th, and 95th. Mean emission changes are over carbon monoxide, nitrogen oxides,

sulphur dioxide, ammonia, black carbon, organic carbon, $PM_{2.5}$, coarse particulate matter, and non−methane volatile organic compounds.

Supplementary Figure 6: Regional changes in exposure and health impacts in China for 2010 to 2020. The bottom−up from Zheng et al., (2018) are for 2010−2017. The top−down estimates from the emulators are for 2015−2020 using the mean of the top 1,000 occurring emission configurations that matched the measured trend in fine particulate matter ($PM_{2.5}$, annual−mean) concentrations only. Results are for (a) $PM_{2.5}$ exposure, (b) annual premature mortalities (MORT) from $PM_{2.5}$ exposure, (c) annual rate of disability−adjusted life years (DALYs) per 100,000 people from $PM_{2.5}$ exposure, (d) ozone (O_3, maximum 6−monthly−mean daily−maximum 8−hour, 6mDM8h) exposure, (e) annual MORT from O_3 exposure, and (f) annual rate of DALYs per 100,000 people from O_3 exposure.

Additional Supporting Information (Files uploaded separately)

The trained emulators per grid cell in China that support the findings of this study are available at doi.org/10.5518/1055.

Supplementary Table 1: The bottom−up emission configurations in China over 2010−2017 relative to 2015 from Zheng et al., (2018). The emission sectors are residential (RES), industry (IND), land transport (TRA), agriculture (AGR), and power generation (ENE). Mean emission changes over carbon monoxide, nitrogen oxides, sulphur dioxide, ammonia, black carbon, organic carbon, fine particulate matter, coarse particulate matter, and non−methane volatile organic compounds.

	**2010**	**2011**	**2012**	**2013**	**2014**	**2015**	**2016**	**2017**
**RES (%)**	115	119	120	113	106	100	92	84
**IND (%)**	127	130	130	129	112	100	84	81
**TRA (%)**	98	101	101	102	99	100	97	99
**AGR (%)**	98	101	102	101	101	100	99	99
**ENE (%)**	136	146	139	129	112	100	94	89

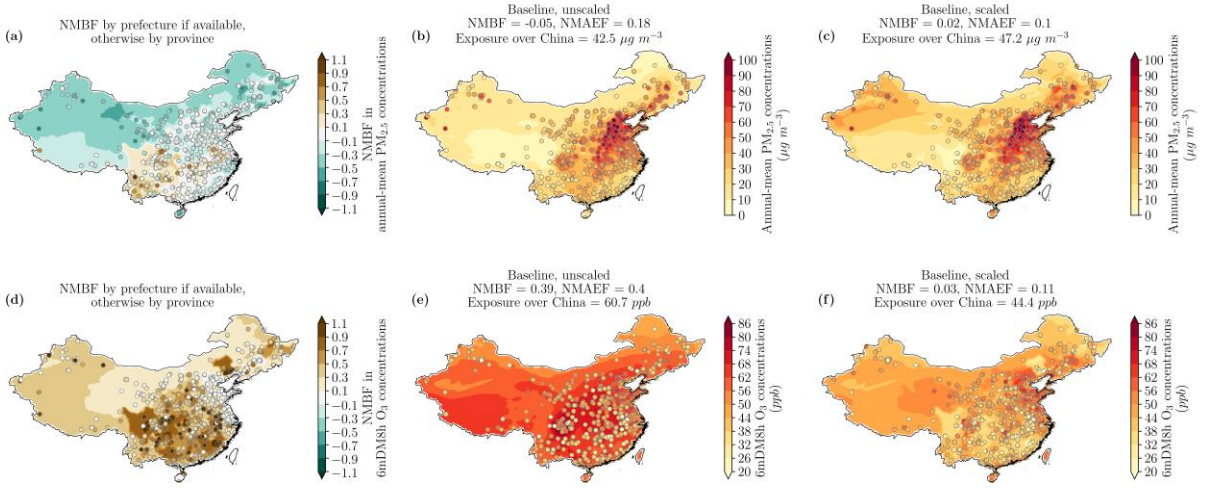

Supplementary Figure 1: Evaluation of unscaled and scaled baseline simulator air quality concentrations (Conibear et al., 2022). Regional evaluation metrics are normalised mean bias factor (NMBF) grouped by prefecture if available, otherwise by province for (a) for fine particulate matter ($PM_{2.5}$, annual−mean) and (d) ozone (O_3, maximum 6−monthly−mean daily−maximum 8−hour, 6mDM8h). Unscaled baseline concentrations for (b) $PM_{2.5}$ (NMBF = −0.05, normalised absolute error factor, NMAEF, = 0.18) and (e) O_3 (NMBF = 0.39, NMAEF = 0.40). Scaled baseline concentrations for (c) $PM_{2.5}$ (NMBF = 0.02, NMAEF, = 0.10) and (f) O_3 (NMBF = 0.03, NMAEF = 0.11).

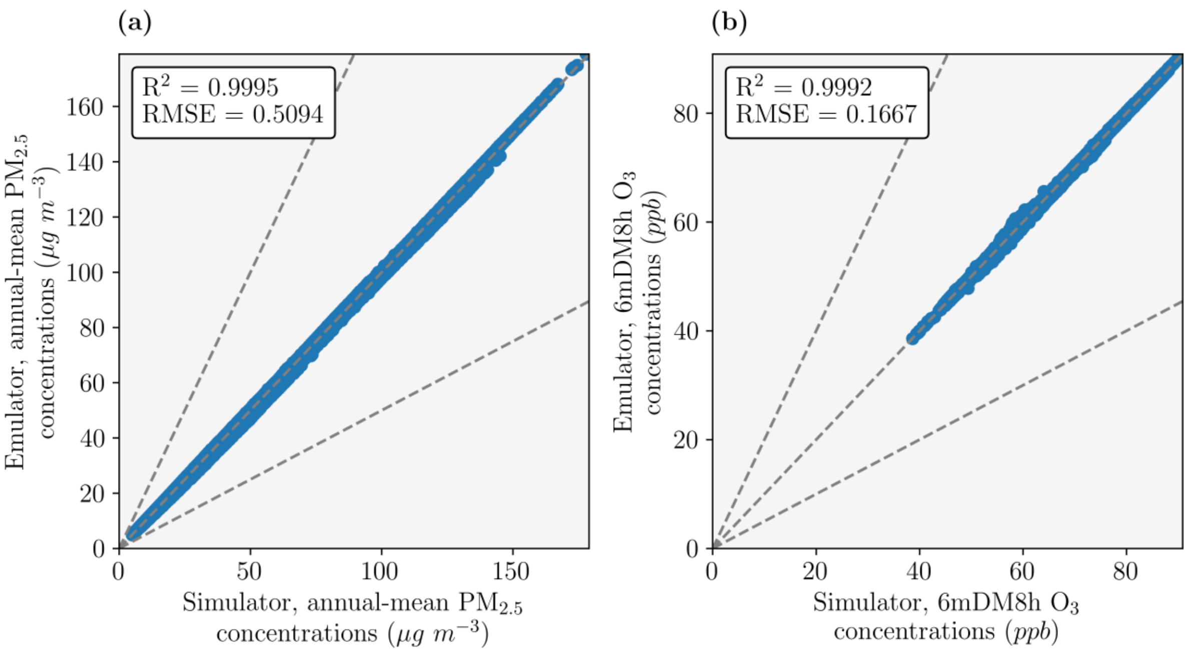

Supplementary Figure 2: Evaluation of emulators on the unseen test data for concentrations of (a) fine particulate matter ($PM_{2.5}$, annual−mean) and (b) ozone (O_3, maximum 6−monthly−mean daily−maximum 8−hour, 6mDM8h) (Conibear et al., 2022). Evaluation metrics used were the coefficient of determination (R^2) and the root mean squared error (RMSE).

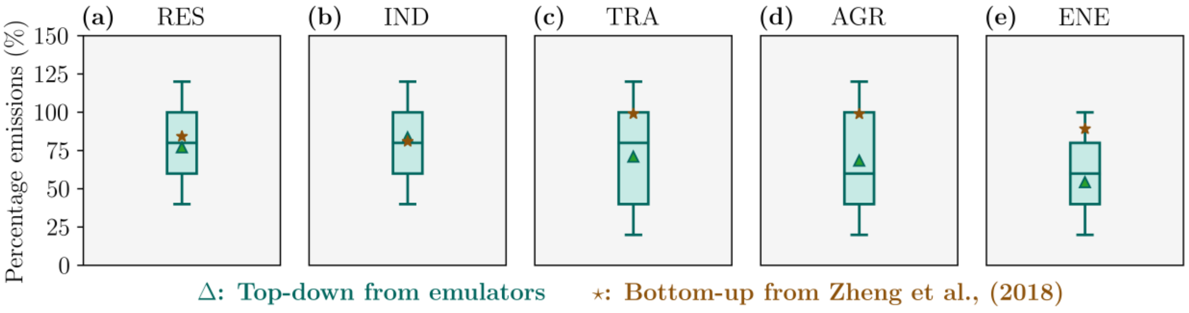

Supplementary Figure 3: Comparison of top−down (emulators) and bottom−up (Zheng et al., 2018) estimates of anthropogenic emission changes in China over 2015−2017. Top−down estimates are for the top 1,000 occurring emission configurations that matched the measured trend *in either fine particulate matter ($PM_{2.5}$, annual−mean) and ozone (O_3, maximum 6−monthly−mean daily−maximum 8−hour, 6mDM8h) concentrations*. Emissions are for the (a) residential (RES), (b) industrial (IND), (c) land transport (TRA), (d) agricultural (AGR), and (e) power generation (ENE) sectors. Boxplot percentiles are 5th, 25th, 50th, 75th, and 95th. Mean emission changes are over carbon monoxide, nitrogen oxides, sulphur dioxide, ammonia, black carbon, organic carbon, $PM_{2.5}$, coarse particulate matter, and non−methane volatile organic compounds.

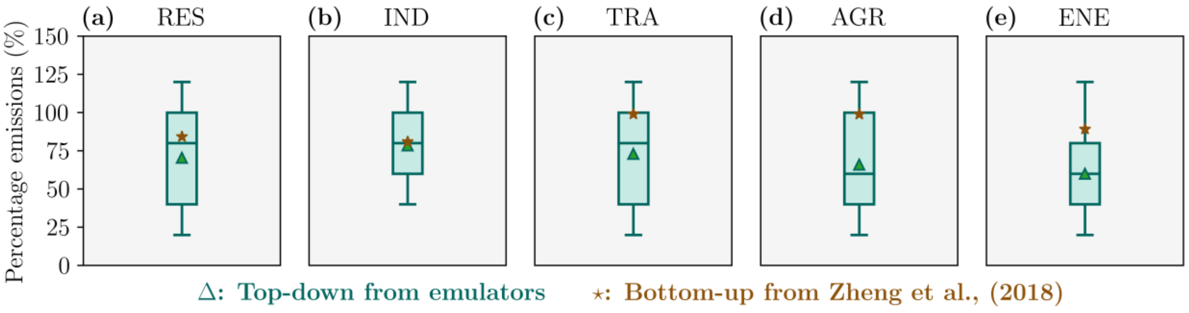

Supplementary Figure 4: Comparison of top−down (emulators) and bottom−up (Zheng et al., 2018) estimates of anthropogenic emission changes in China over 2015−2017. Top−down estimates are from the top 1,000 occurring emission configurations that match the measured trend in *both fine particulate matter ($PM_{2.5}$,*

annual−mean) and ozone (O_3, maximum 6−monthly−mean daily−maximum 8−hour, 6mDM8h) concentrations. Emissions are for the (a) residential (RES), (b) industrial (IND), (c) land transport (TRA), (d) agricultural (AGR), and (e) power generation (ENE) sectors. Boxplot percentiles are 5th, 25th, 50th, 75th, and 95th. Mean emission changes are over carbon monoxide, nitrogen oxides, sulphur dioxide, ammonia, black carbon, organic carbon, $PM_{2.5}$, coarse particulate matter, and non−methane volatile organic compounds.

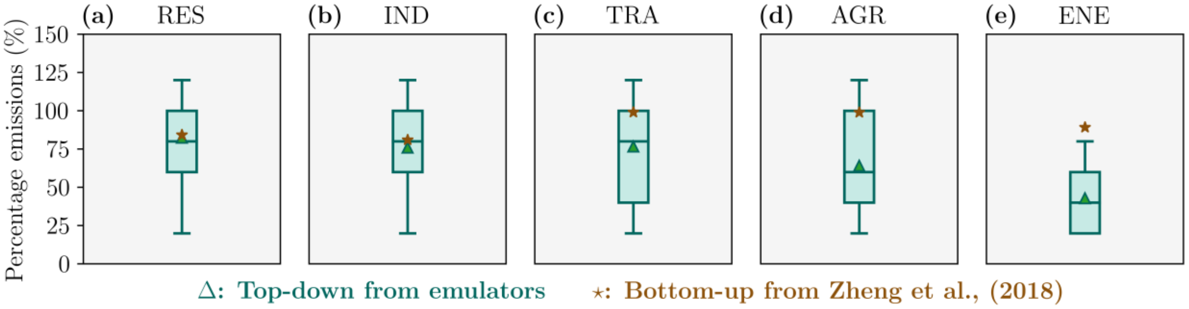

Supplementary Figure 5: Comparison of top−down (emulators) and bottom−up (Zheng et al., 2018) estimates of anthropogenic emission changes in China over 2015−2017. Top−down estimates are for the top 1,000 occurring emission configurations that matched the measure*d trend in ozone (O_3, maximum 6−monthly−mean daily−maximum 8−hour, 6mDM8h) concentrations only*. Emissions are for the (a) residential (RES), (b) industrial (IND), (c) land transport (TRA), (d) agricultural (AGR), and (e) power generation (ENE) sectors. Boxplot percentiles are 5th, 25th, 50th, 75th, and 95th. Mean emission changes are over carbon monoxide, nitrogen oxides, sulphur dioxide, ammonia, black carbon, organic carbon, $PM_{2.5}$, coarse particulate matter, and non−methane volatile organic compounds.

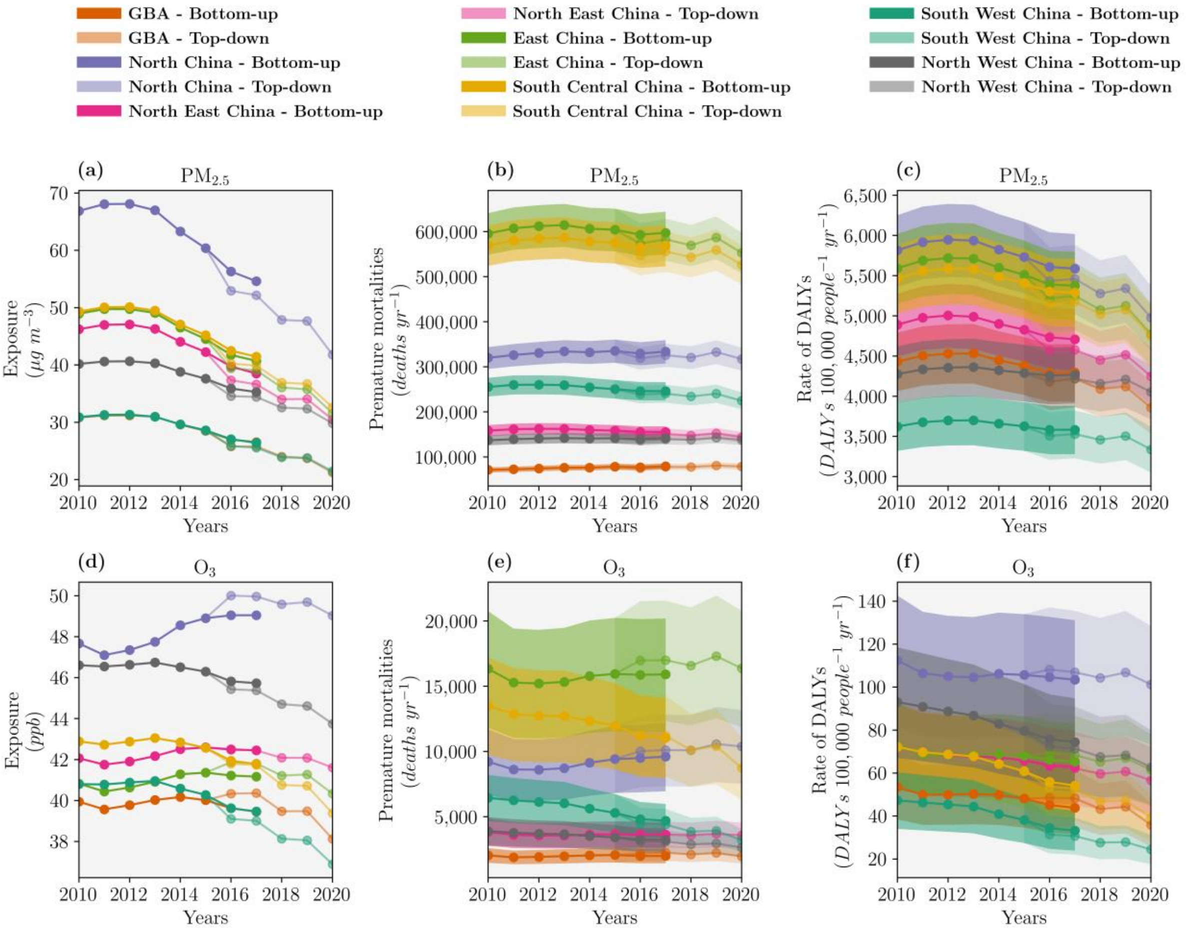

Supplementary Figure 6: Regional changes in exposure and health impacts in China for 2010 to 2020. The bottom−up from Zheng et al., (2018) are for 2010−2017. The top−down estimates from the emulators are for 2015−2020 using the mean of the top 1,000 occurring emission configurations that matched the measured trend

in fine particulate matter ($PM_{2.5}$, annual−mean) concentrations only. Results are for (a) $PM_{2.5}$ exposure, (b) annual premature mortalities (MORT) from $PM_{2.5}$ exposure, (c) annual rate of disability−adjusted life years (DALYs) per 100,000 people from $PM_{2.5}$ exposure, (d) ozone (O_3, maximum 6−monthly−mean daily−maximum 8−hour, 6mDM8h) exposure, (e) annual MORT from O_3 exposure, and (f) annual rate of DALYs per 100,000 people from O_3 exposure.

References

Conibear, L., Reddington, C. L., Silver, B. J., Chen, Y., Knote, C., Arnold, S. R., et al. (2022). Sensitivity of Air Pollution Exposure and Disease Burden to Emission Changes in China using Machine Learning Emulation. *GeoHealth*. https://doi.org/10.1029/2021GH000570

Zheng, B., Tong, D., Li, M., Liu, F., Hong, C., Geng, G., et al. (2018). Trends in China's anthropogenic emissions since 2010 as the consequence of clean air actions. *Atmospheric Chemistry and Physics*, *18*(19), 14095–14111. https://doi.org/10.5194/acp-18-14095-2018
